# Incidence and predictive factors for development of ADHD post congenital heart disease surgery—a single tertiary center experience from India

**DOI:** 10.3389/fcvm.2026.1634911

**Published:** 2026-04-23

**Authors:** Akriti Gera, Amit Misri, Pankaj Bajpai, Shefali Yadav

**Affiliations:** Department of Pediatric Cardiology and Congenital Heart Disease, Medanta Hospital, Gurugram, Haryana, India

**Keywords:** ADHD, ADHD (attention deficit and hyperactivity disorder), cardiac surgery, congenital heart disease, neurodevelopment

## Abstract

**Introduction:**

A recent shift in mortality trends in children with congenital heart disease (CHD) has led to more patients reaching adulthood. Attention-deficit hyperactivity disorder (ADHD) is a prevalent and often underdiagnosed condition, with increased risk observed in children with CHD due to disease- and surgery-related factors. This study aimed to determine the incidence of ADHD one year after cardiac surgery in children and to identify associated clinical and operative risk factors.

**Methods:**

Children aged 3-5 years and 6-18 years who were to undergo cardiac surgery for CHD between December 2022 to June 2023, were evaluated pre-operatively and post-operatively ≥1 year after the surgery, after informed written consent.

**Results:**

Our study comprised of 98 children. The incidence of ADHD in children 1 year post cardiac surgery was 32.6%. We also found a statistically significant association between post- operative complications, longer CPB time, aortic cross-clamp time, duration of mechanical ventilation, ICU stay, and hospital stay and ADHD. No significant association was found with prior cardiac surgery, cyanosis, intraoperative complications, reintervention, or inotrope duration. CPB time >58 minutes, aortic cross-clamp time >46 minutes, hospital stay >8 days, and ICU stay ≥3 days demonstrated moderate to good predictive accuracy for ADHD.

**Conclusion:**

ADHD is a common outcome following pediatric cardiac surgery and is associated with perioperative factors, particularly markers of surgical complexity and postoperative recovery. Early identification using these predictors may help reduce long-term morbidity, though larger studies are needed to validate these findings.

## Introduction

Congenital heart disease (CHD) is among the most prevalent congenital anomalies, afflicting 8–12 per 1,000 live births worldwide (1). Considering a rate of 9/1,000, about 1.35 million babies are born with CHD each year globally (2). For India, this translates to approximately 240,000 affected children per year (3). Owing to advancements made in medical technology and surgical techniques, there has been a shift in mortality trends in patients with CHDs (4). More than 90% of children born with CHD reach adulthood (5) and mortality rates have decreased by 31% over the last 20 years (4). As a result, the research focus has progressively shifted from improving survival to minimizing long-term morbidities associated with CHD and its treatment.

Attention-deficit hyperactivity disorder (ADHD) is a common childhood behavioral disorder. According to Salari et al. (6), the incidence of ADHD is 7.6% in children aged 3 to 12 years and 5.6% in teenagers aged 12 to 18. Systematic reviews indicate that the community prevalence globally is between 2% and 7%, with an average of around 5% (5–10). However, ADHD remains under-recognized and underdiagnosed in many populations. Children with ADHD have poorer long-term outcomes with respect to academic achievement and attainment, occupational rank and job performance, risky sexual practices and early unwanted pregnancies, substance use, relationship difficulties, marital problems, traffic violations, and car accidents (11). Given these substantial consequences, early diagnosis and intervention are critical.

One of the most pressing parental concerns is the neurodevelopmental outcome of their child.

Children with CHD are at a 30% higher risk for inattention and hyperactivity compared to healthy individuals (5). The abnormal cerebral blood flow, which is altered antenatally and perinatally, potentially causes delays in brain development. Abnormal sulcal folding patterns have also been noted in adults with simple CHDs (ASD/VSD) (12).

Despite medical advancements, CHD and its’ management is not completely foolproof. Pediatric cardiologists and cardiac surgeons are hesitant with early repair, whether definitive or palliative, due to the multi-organ effect of cardiac surgery. Additional hypoxaemia, due to postponed correction of cardiac defects, seems to increase the risk for attentional dysfunction, hyperactivity and is considered responsible for further damage to the highly oxygen-sensitive regions of the prefrontal cortex (13). Unfortunately, there are no consensus guidelines on the neuroprotective strategies to be followed during surgeries. Studies have shown that the risk of developing ADHD in adolescence is significantly higher in cardiac surgery patients compared to healthy individuals. The symptoms may worsen by repeated exposure to anaesthetics and surgery-induced stress and inflammatory responses. Single, or repeated anaesthesia could be a risk factor for development of ADHD symptoms, independent of the surgery performed (14–16).

### Study objectives

In this study, we aimed to assess the incidence of ADHD one year following cardiac surgery in children aged 3–5 years (using the ADHD Rating Scale-IV, Preschool Version) and 6–18 years (using the Conners 3 Short Form—Parent version). Additionally, we sought to identify clinical and operative predictors associated with the development of ADHD symptoms in this population.

## Methodology

Following approval from the institutional ethics committee, this prospective observational study was conducted at Medanta Hospital, Gurugram, Haryana, India, over a period of 18 months (December 2022—June 2024). The study included pediatric patients aged 3–5 years and 6–18 years who were scheduled to undergo cardiac surgery for congenital heart disease (CHD) between December 2022 and June 2023.

### Exclusion criteria

The exclusion criteria were children with known case of neuropsychiatric illness or receiving treatment for the same, genetic syndromes associated with cognitive deficits (eg: Downs syndrome), multiple congenital anomalies, severe neurological impairment, lack of perioperative data and lack of parental or primary care giver's consent.

### Preoperative assessment

Eligible participants were assessed both preoperatively and at least one year postoperatively, following informed written consent from parents or primary caregivers. Baseline demographic and clinical data were collected, including age, sex, vital signs, cardiac examination findings, echocardiographic diagnosis, and history of previous cardiac surgeries.

ADHD screening was conducted preoperatively. Children who screened negative were enrolled in the study. For children aged 3–5 years, the ADHD Rating Scale IV—Preschool Version was completed by the parent or primary caregiver. For those aged 6–18 years, the Conners' Parent Rating Scale–Short Form was used. Self-report was not included due to developmental variability in insight and reliability across the 6–18-year age range, and to ensure feasibility and consistency in the hospital setting.

### Intraoperative data collection

Intraoperative variables documented included the type of surgical procedure performed, durations of cardiopulmonary bypass (CPB), aortic cross-clamp (ACC), and deep hypothermic circulatory arrest (DHCA), the lowest temperature during CPB, and any intraoperative complications along with their management.

### Postoperative data collection

Postoperative outcomes assessed were duration of ICU stay, total hospital stay, duration of mechanical ventilation, inotropic support, need for extracorporeal membrane oxygenation (ECMO), requirement for reintervention, and the occurrence and management of postoperative complications such as hypoglycemia, seizures, or cardiac arrest.

### Follow-up and ADHD assessment

At least one year following surgery, all participants underwent reassessment using age-appropriate, validated ADHD screening questionnaires during their routine follow-up outpatient visits. These assessments were conducted by trained pediatricians. Children with scores indicative of ADHD were referred to a child psychiatrist for confirmatory diagnosis and further management. However, psychiatric diagnostic outcomes were not systematically captured within the study database, as the study was designed to evaluate screening results rather than establish formal psychiatric diagnoses.

### Sample size calculation

The primary objective of the study was to determine the incidence, i.e., the development of ADHD after 1 year of cardiac surgery. The likely value of incidence (i) is not known for the Indian subcontinent. In such situations, a sample size of 96 was recommended for estimating the incidence within 10% margin of error and with 95% confidence level.

### Data management and statistical analysis

The analysis involved profiling patients based on demographic characteristics, clinical parameters (both preoperative and postoperative), and surgical findings. Descriptive statistics for quantitative variables were reported as means with standard deviations, while categorical variables were presented as absolute numbers and percentages. Comparative analyses were conducted between patients who developed ADHD and those who did not, examining various preoperative, postoperative, and background parameters. Independent samples *t*-tests were used to compare means between groups. Cross-tabulations were created for categorical variables, and associations were tested using the Chi-square test. Univariate logistic regression analysis was performed to assess the relationship between demographic, clinical, and surgical variables and the risk of developing ADHD. Variables found to be statistically significant in the univariate analysis were further included in a multivariate logistic regression model to identify independent predictors. A *p value* of <0.05 was considered statistically significant. All statistical analyses were performed using IBM SPSS Statistics for Windows, Version 23.0 (IBM Corp., Armonk, NY).

### Ethical issues

#### Consent, scientific and ethical review

This study was initiated after clearance from the institutional ethical committee. Each subject has been enrolled with informed written consent.

#### Confidentiality

Patient records have been kept confidential and anonymous. Patients who opted to withdraw consent during the study period have continued to receive standard care as per the hospital practice.

## Results

Our study population comprised of 98 children, whose pre-operative parameters and complications are described in Table 1.

**Table 1:** Distribution of all pre-operative parameters and complications in study group.

Variable	Number of patients (*n* = 98)		Percentage (%)
Gender			
Female	38		38.8%
Male	60		61.2%
History of Cardiac Surgery			
Yes	33		33.7%
	CPB (+)	26	78.8%
	CPB (−)	7	21.2%
No	65		66.3%
Type of CHD			
Acyanotic	40		40.8%
Cyanotic	58		59.2%
Intra-operative complications			
Yes	6		6.1%
No	92		93.9%
Reintervention			
Yes	4		4.1%
No	94		95.9%
Post operative complications			
Yes	46		47.0%
No	52		53.0%
If yes (*n* = 46)			
Rhythm Abnormalities	20		43.5%
Hypertension	8		17.4%
Hemoptysis- Coiling	2		4.3%
Acute kidney injury	1		2.2%
Prolonged Pleural Drain (>3 days)	6		13%
Post-periocardiotomy Syndrome	3		6.5%
Low cardiac output state	2		4.3%
Prolonged oxygen support (8 days)	2		4.3%
Thrombectomy	2		4.3%

The mean age at surgery was 7.9 ± 4.1 years. 10 patients underwent closed heart surgery. 18 patients went on CPB but did not undergo aortic cross clamping. Intra- operatively, the mean bypass time was 63.6 ± 21.3 min and aortic cross-clamp time was 46.9 ± 16.9 min. All patients were cooled to 34.4 ± 1.5°Celsius and only one patient was cooled to 25 degrees. None of the patients in the study group underwent DHCA. Post operatively, the mean duration on the mechanical ventilator was 16.7 ± 9.8 h. Mean duration of inotrope requirement was 2.5 ± 1.1 days. The mean duration of hospital stay was 8.7 ± 2.1 days, which included ICU stay, the mean duration of which was 2.8 ± 1.1 days.

### Incidence of ADHD post cardiac surgery

The ADHD questionnaire was performed 13.5 ± 1.2 months from surgery. The incidence of ADHD post cardiac surgery in our study was 32.6% (Table 2).

**Table 2:** ADHD questionnaire.

ADHD Questionnaire	Number of patients	Percentage (%)
ADHD Rating scale IV—Preschool	18	18.4%
Connors	80	81.6%
Interpretation		
Inattentive	22	22.4%
Hyperactive + Impulsive	10	10.2%
Nil	66	67.3%

### Preoperative clinical parameters and ADHD test result

There was no statistically significant association between preoperative clinical parameters (gender, history of cardiac surgery, and type of CHD) with positive ADHD test result (*p* value >0.05) (Table 3). The mean age group of patients who tested positive for ADHD was 8.6 ± 4.2 years, while those who tested negative had a mean age group of 7.6 ± 4 years. The difference in age group was not statistically significant; hence the groups were demographically similar in age.

**Table 3:** Association of pre-operative clinical parameters with ADHD test result.

Parameter		Positive		Negative		Total		Chi square Value	*p*—value
		*n*	%	*n*	%	*n*	%		
Gender	Female	10	26.3%	28	73.7%	38	100.0%	1.133	0.287
	Male	22	36.7%	38	63.3%	60	100.0%		
H/O cardiac surgery	No	18	27.7%	47	72.3%	65	100.0%	2.160	0.142
	Yes	14	42.4%	19	57.6%	33	100.0%		
Type of CHD	Acyanotic	9	22.5%	31	77.5%	40	100.0%	3.168	0.075
	Cyanotic	23	39.7%	35	60.3%	58	100.0%		

**Figure 1:**
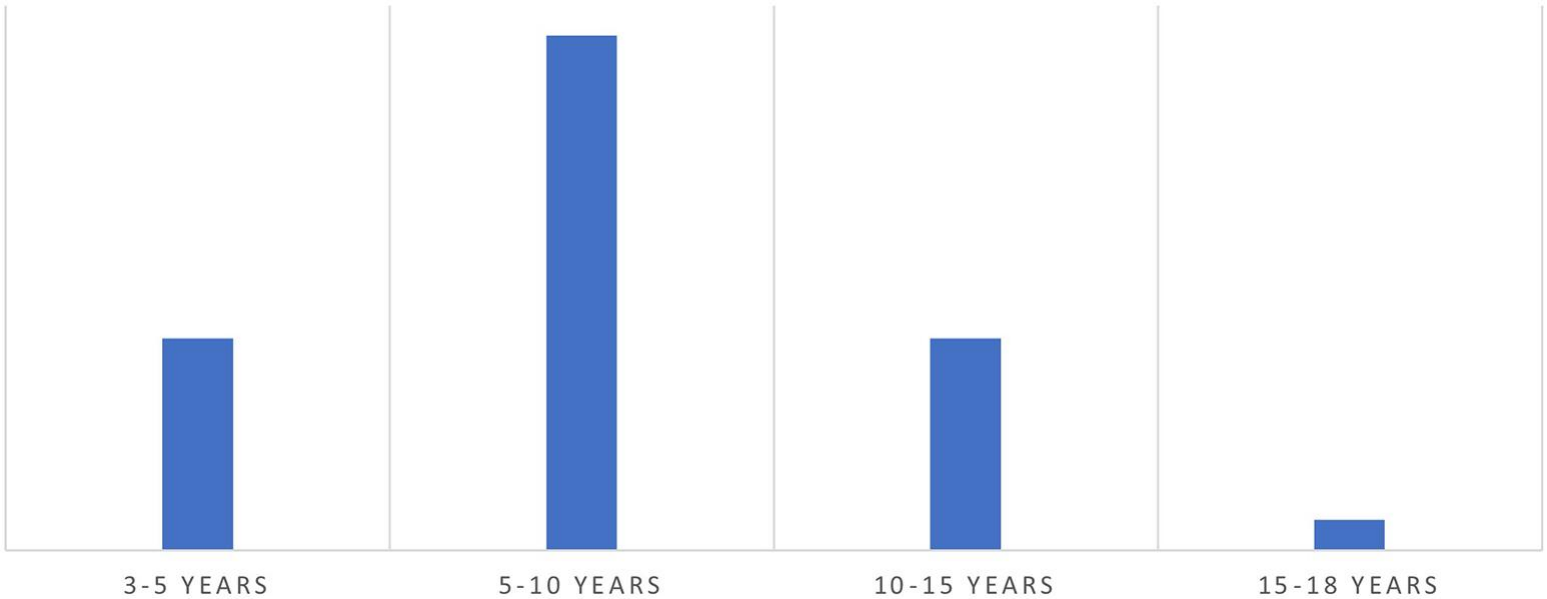
Age wise distribution of children who tested ADHD Positive.

Figure 1 shows the age wise distribution of children who tested positive for ADHD.

### Complications and ADHD test result

We did not find a statistically significant difference between intra-operative complications or need of re-interventon with ADHD test result (*p* value >0.05 in both) (Table 4). However, 68.1% of those who did not require reintervention tested negative for ADHD. We also found a statistically significant association (*p* value 0.045) between presence of post- operative complications and positive test result of ADHD questionnaire.

**Table 4:** Association of complications with respect ADHD test result.

Parameter		Positive		Negative		Total		Chi Squa re Value	*p*—value
		*n*	%	*n*	%	*n*	%		
Intra-op. Complications	No	29	31.5%	63	68.5%	92	100.0%	0.875	0.350
	Yes	3	50.0%	3	50.0%	6	100.0%		
Reintervention	No	30	31.9%	64	68.1%	94	100.0%	0.571	0.450
	Yes	2	50.0%	2	50.0%	4	100.0%		
Post op. Complications	No	12	23.5%	39	76.5%	51	100.0%	4.025	0.045
	Yes	20	42.6%	27	57.4%	47	100.0%		

When further explored, we calculated that those with post-operative complication were more likely to test as inattentive and 76.4% of children without post-operative tested negative for the ADHD questionnaire (Table 5).

**Table 5:** Post-operative complications and ADHD questionnaire score.

Parameter		Inattentive		Hyperactive + Impulsive		Nil		Total	
		*N*	%	*N*	%	*N*	%	*N*	%
Post operative complication	No	11	21.5%	1	1.96%	39	76.4%	51	100.0%
	Yes	11	23.4%	9	19.2%	27	57.4%	47	100.0%

### Operative and ICU parameters and ADHD test result

**Figure 2:**
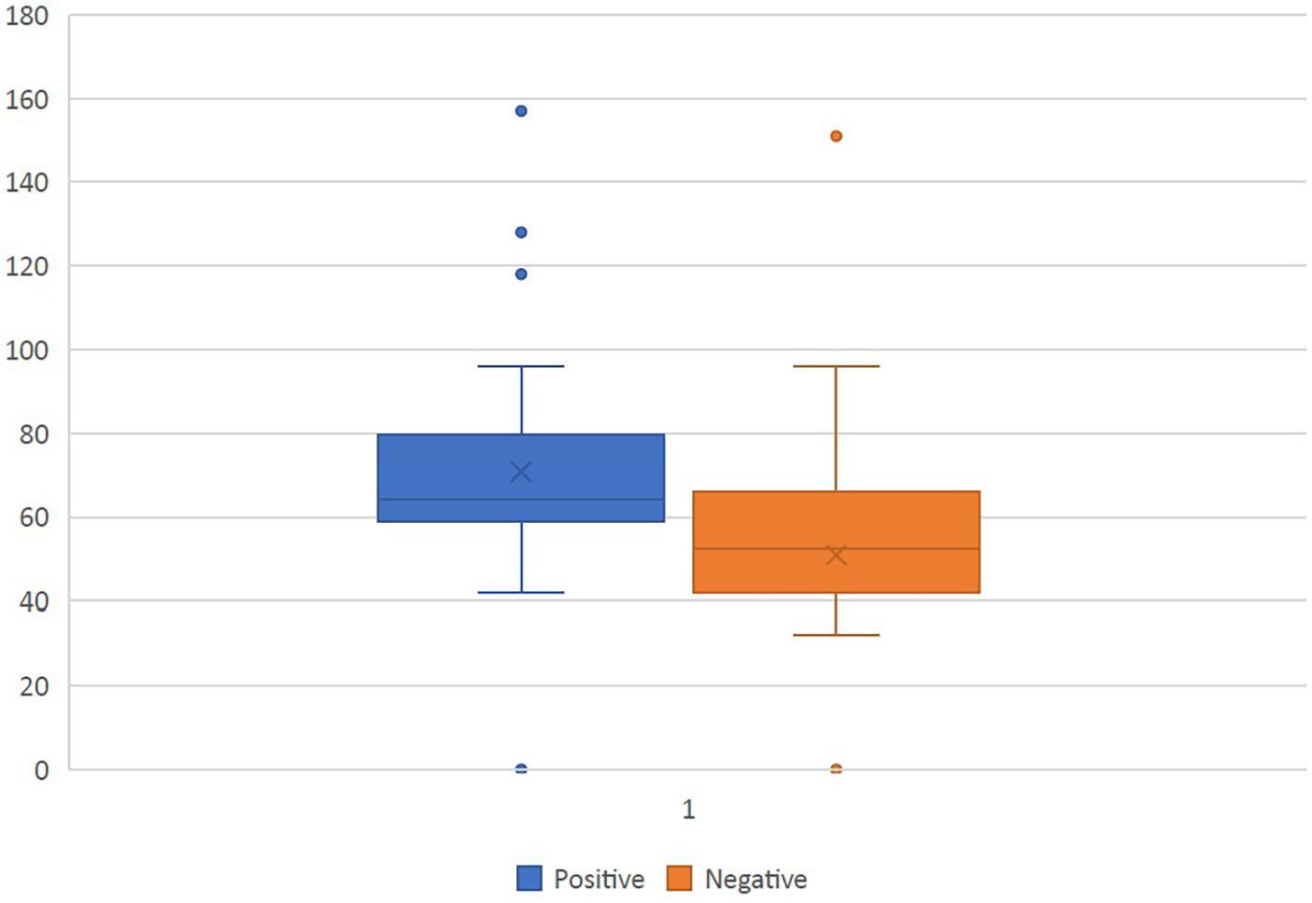
Box and whisker plot representation of CPB time and ADHD questionnaire.

There was a statistically significant association between duration of cardiopulmonary bypass, aortic cross clamp time, MV duration, hospital stay (ICU + ward stay) with a positive outcome on the ADHD questionnaire (Table 6). On subset analysis of hospital stay, we found that the duration of ICU stay had a statistically significant association with positive ADHD test result (*p* value 0.002). Figure 2 shows box and whisker plot representation of CPB Time and ADHD Questionnaire.

**Table 6:** Comparison of mean value of operative and ICU parameters with ADHD test result.

Parameter	Positive mean ± SD	Negative mean ± SD	Mean ± Std err of the difference	95% confidence interval of the difference		*t*- value	*p* value
				Lower	Upper		
Bypass Time (minutes)	73.1 ± 25	59.1 ± 17.7	14 ± 4.4	5.2	22.8	3.164	0.002
Aortic CC time	60.6 ± 21.9	41.9 ± 11.4	18.6 ± 3.8	11.1	26.2	4.924	<0.001
MV (hours)	20 ± 9.8	15.1 ± 9.4	4.8 ± 2.1	0.8	8.9	2.350	0.021
Inotropes (days)	2.6 ± 1.1	2.4 ± 1.1	0.2 ± 0.2	*−0*.*3*	0.6	0.736	0.464
Hospital Stay (days)	9.9 ± 2.3	8 ± 1.6	1.9 ± 0.4	1.1	2.7	4.620	<0.001
ICU stay (days)	3.3 ± 1.3	2.5 ± 0.9	0.7 ± 0.2	0.3	1.1	3.199	0.002
Time since surgery (months)	13.3 ± 1	13.6 ± 1.3	−0.3 ± 0.3	*−0.8*	0.2	−1.071	0.287

Our data revealed that the children who had a longer CPB time, longer aortic cross-clamp time and longer hospital stay were more likely to test as inattentive (Table 7). Additionally, those who had a longer duration on MV and ICU stay were more likely to test as hyperactive + impulsive (Table 7). It is important to note that the mechanical ventilation duration corresponds to the duration in ICU, and not during cardiac surgery.

**Table 7:** Comparison of mean value of statistically significant parameters with ADHD test result.

Parameter	Inattentive	Hyperactive + Impulsive	Nil	F—value	*p*—value
Bypass Time (minutes)	79.2 ± 27.5	60.4 ± 11.5	59.1 ± 17.7	8.327	<0.001
Aortic CC time	64.7 ± 24.7	50.2 ± 5.3	41.9 ± 11.4	14.790	<0.001
MV (hours)	18.2 ± 4.3	23.8 ± 16.3	15.1 ± 9.4	3.988	0.022
Hospital Stay (days)	10 ± 2.6	9.7 ± 1.8	8 ± 1.6	10.670	<0.001
ICU stay	3.1 ± 1.2	3.6 ± 1.5	2.5 ± 0.9	6.013	0.003

**Figure 3:**
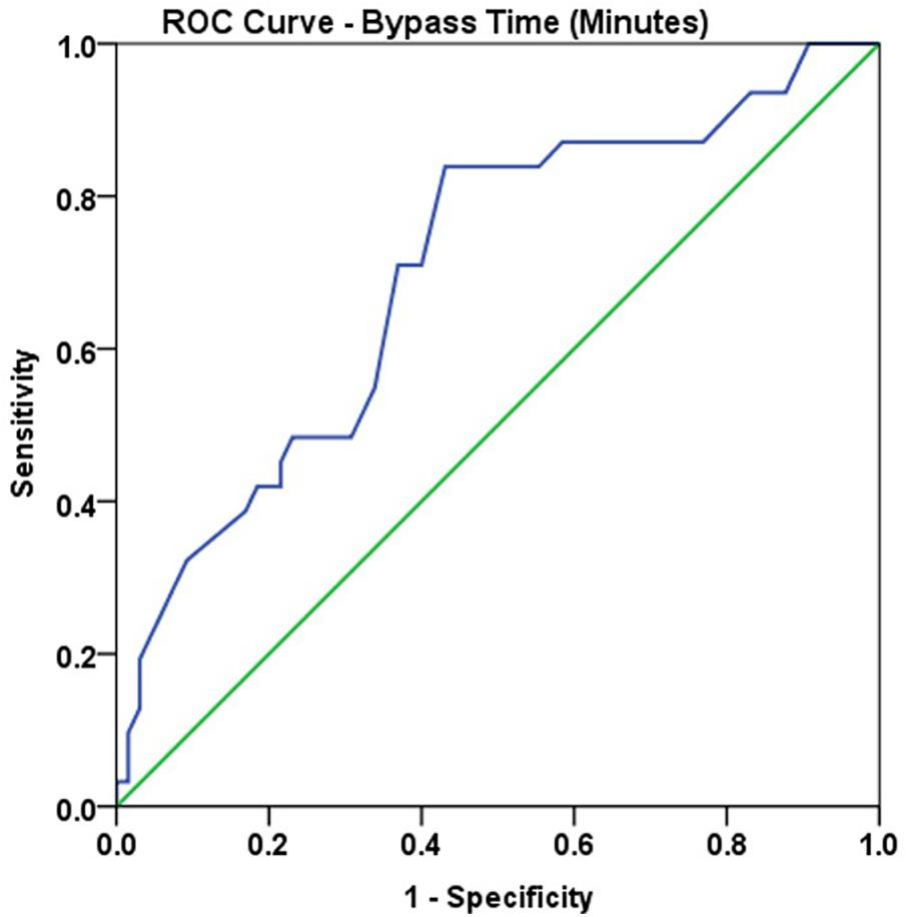
ROC curve: CPB time (min.) and ADHD Positive.

Using ROC analysis, CPB time of >58 min showed a good accuracy to predict ADHD positive, with sensitivity of 83.9% and specificity of 56.9% (Figure 3, Tables 8, 9).

**Table 8:** ROC analysis: CPB time and ADHD positive.

Parameter	AUC	Std. Error	Asymptotic 95% confidence interval		*p*—value
			Lower bound	Upper bound	
CPB Time (minutes)	0.701	0.057	0.589	0.813	0.002

**Table 9:** Optimal Cut-off point: CPB time and ADHD POSITIVE.

Parameter	Optimal cut-off point	Sensitivity	Specificity
CPB Time (minutes)	58	83.9%	56.9%

**Figure 4:**
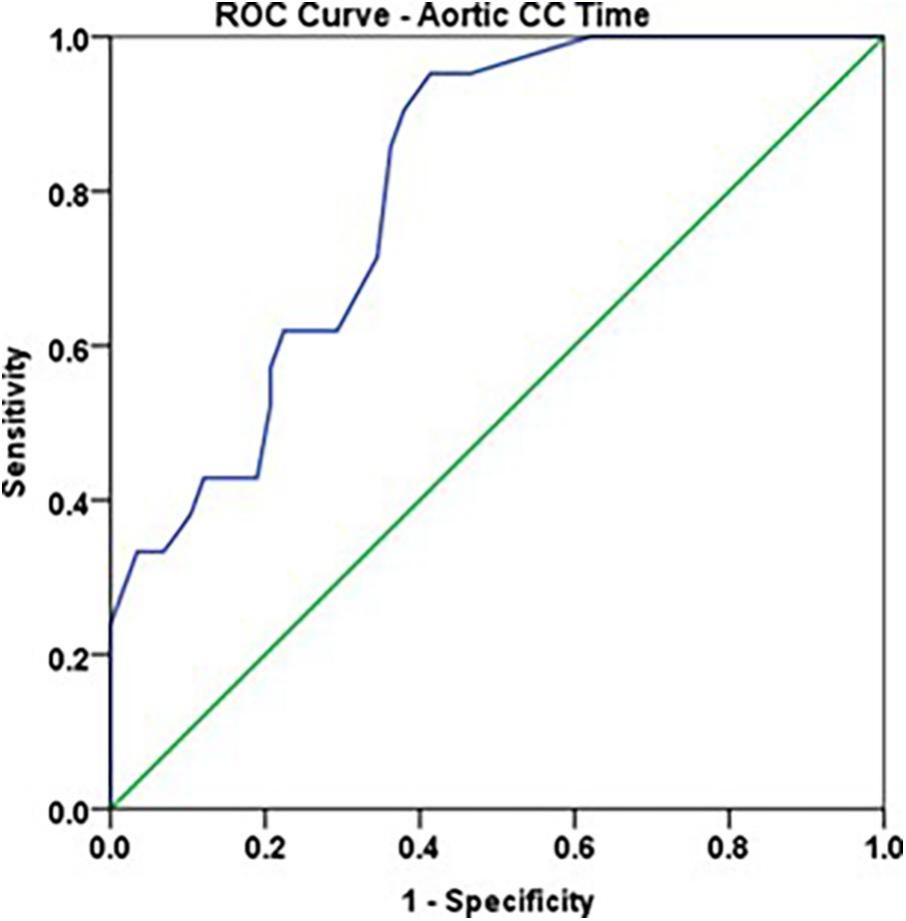
ROC curve: aortic CC time (min.) and ADHD Positive.

Aortic cross clamp time of >46 min showed a very good accuracy to predict ADHD positive, with sensitivity of 85.7% and specificity of 63.8% (Figure 4, Tables 10, 11).

**Table 10:** ROC analysis: aortic CC time and ADHD POSITIVE.

Parameter	AUC	Std. Error	Asymptotic 95% confidence interval		*p*—value
			Lower bound	Upper bound	
Aortic CC Time (minutes)	0.807	0.049	0.710	0.904	<0.001

**Table 11:** Optimal Cut-off point: aortic CC time and ADHD POSITIVE.

Parameter	Optimal cut-off point	Sensitivity	Specificity
Aortic CC Time (minutes)	46	85.7%	63.8%

**Figure 5:**
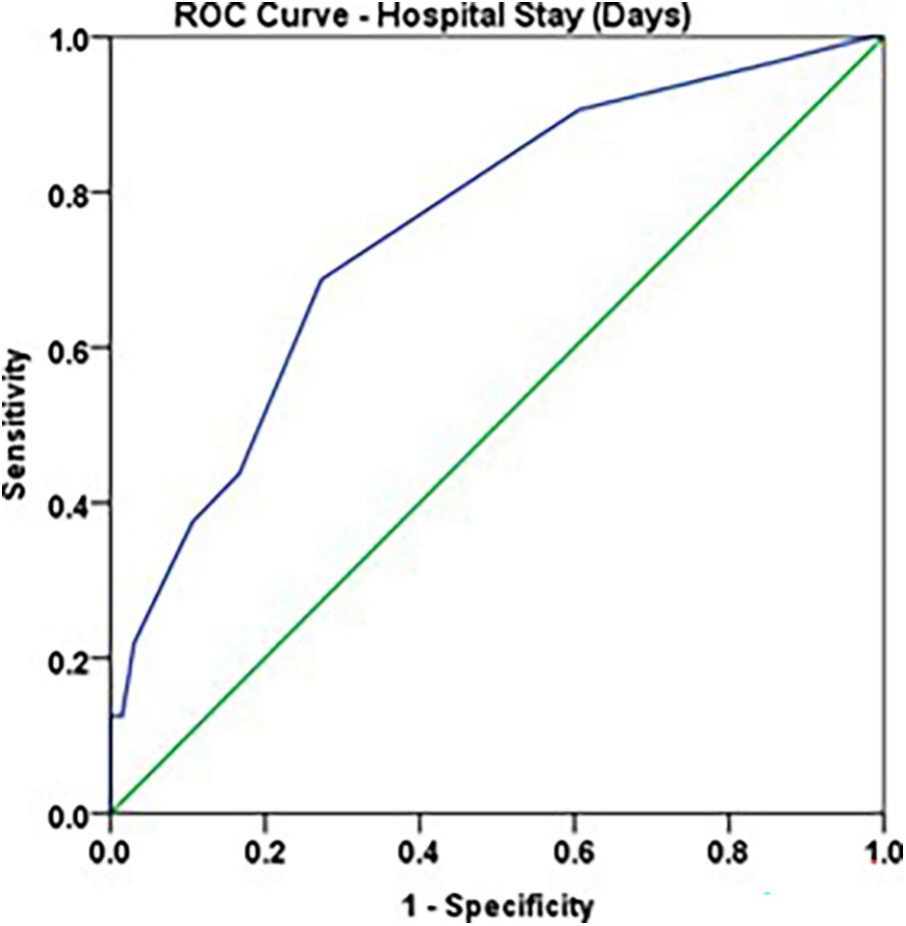
ROC curve: hospital stay (>8 days) and ADHD Positive.

Duration of hospital stay >8 days showed a good accuracy to predict ADHD positive, with sensitivity of 68.8% and specificity of 72.7% (Figure 5, Tables 12, 13).

**Table 12:** ROC analysis: hospital stay and ADHD POSITIVE.

Parameter	AUC	Std. Error	Asymptotic 95% Confidence Interval		*p*—value
			Lower bound	Upper bound	
Hospital Stay (days)	0.753	0.052	0.651	0.855	<0.001

**Table 13:** Optimal cut-off point: aortic CC time and ADHD POSITIVE.

Parameter	Optimal cut-off point	Sensitivity	Specificity
Hospital Stay (days)	8	68.8%	72.7%

**Figure 6:**
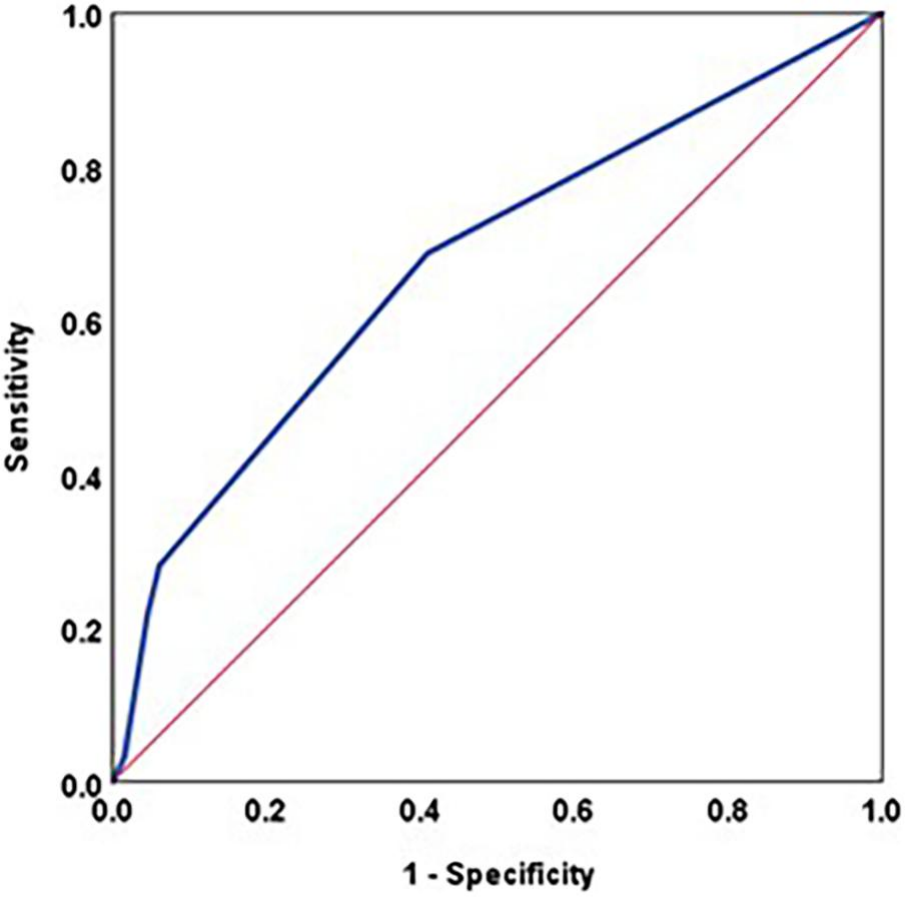
ROC curve: ICU stay (days) and ADHD Positive.

Since hospital stay included duration of ICU stay and ward stay, we further explored this finding for ICU stay specifically. Duration of ICU stay ≥3 days showed a fair accuracy to predict ADHD positive, with sensitivity of 68.8% and specificity of 59.1% (Figure 6, Tables 14, 15).

**Table 14:** ROC analysis: ICU stay and ADHD POSITIVE.

Parameter	AUC	Std. Error	Asymptotic 95% confidence interval		*p*—value
			Lower bound	Upper bound	
ICU Stay (days)	0.675	0,060	0.558	0.793	0.005

**Table 15:** Optimal cut-off point: ICU stay and ADHD POSITIVE.

Parameter	Optimal cut-off point	Sensitivity	Specificity
ICU Stay (days)	≥3	68.8%	59.1%

As seen in Table 16, in the univariate logistic regression analysis, age at the time of surgery was the only significant predictor of ADHD**,** with younger age strongly associated with increased risk (OR = 0.03, 95% CI: 0–0.46, *p* = 0.011). All other examined factors—including gender, history of previous cardiac surgery, type of CHD, hospital stay, bypass time, aortic cross-clamp time, intra- and post-operative complications, mechanical ventilation, inotropes, ICU stay, and time since surgery—did not show statistically significant associations, as their *p*-values exceeded 0.05 and confidence intervals were wide.

**Table 16:** Univariate logistic regression for prediction of ADHD.

Variable	Beta coefficient	Std Err	Odds ratio	95% C.I.for odds ratio		*p*-value
				Lower	Upper	
Age at time of surgery (years)	−3.41	1.34	0.03	0	0.46	0.011
Gender (RC: Female)	0.6	0.57	1.83	0.59	5.61	0.294
HO previous cardiac surgery	−1.1	0.67	0.33	0.09	1.25	0.103
(RC: Acyanotic)	0.7	0.57	2.02	0.66	6.21	0.219
Hospital Stay (days)	−0.12	0.14	0.89	0.67	1.17	0.392
Bypass Time (minutes)	0	0.01	1	0.98	1.02	0.968
Aortic CC time	−0.01	0.02	0.99	0.95	1.03	0.535
Cooled to	−0.04	0.2	0.96	0.65	1.43	0.841
Intra-op Complications	0.86	0.91	2.37	0.4	14.1	0.341
Re-exploration	−19.76	20,096.49	0	0		0.999
MV (hours)	−0.02	0.03	0.98	0.91	1.04	0.475
Inotropes	−0.18	0.27	0.84	0.49	1.44	0.52
ICU stay	−0.2	0.28	0.82	0.47	1.42	0.472
Post-operative complications	0.37	0.52	1.45	0.52	4.06	0.477
Time since surgery (months)	−0.33	0.23	0.72	0.45	1.14	0.158

In the multivariate logistic regression model (Table 17), age at the time of surgery emerged as the only independent predictor of ADHD, with younger age significantly increasing the odds (OR = 0.03, 95% CI: 0–0.36, *p* = 0.005). All other perioperative and postoperative variables, including gender, history of previous cardiac surgery, CHD type, hospital stay, bypass time, aortic cross-clamp time, intraoperative and postoperative complications, mechanical ventilation, inotropes, ICU stay, and time since surgery, did not show statistically significant associations after adjustment. The wide confidence intervals observed for several predictors suggest instability of estimates, likely due to sample size limitations.

**Table 17:** Multivariate logistic regression for prediction of ADHD.

Variable	Beta coefficient	Std Err	Odds ratio	95% C.I.for odds ratio		*p*-value
				Lower	Upper	
Age at time of surgery (years)	−3.51	1.26	0.03	0	0.36	0.005
Gender (RC: Female)	2.08	4.45	7.98	0	49,193.03	0.641
HO previous cardiac surgery	0.51	4.71	1.67	0	16,956.23	0.914
(RC: Acyanotic)	3.87	5.48	47.78	0	22,12,315.33	0.481
Hospital Stay (days)	−0.43	1.2	0.65	0.06	6.88	0.721
Bypass Time (minutes)	0.8	0.56	2.22	0.74	6.66	0.154
Aortic CC time	−0.86	0.62	0.42	0.13	1.42	0.164
Cooled to	−1.49	4.06	0.23	0	647.56	0.714
Intra-op Complications	−0.86	14.77	0.42	0	1.58953E + 12	0.954
MV (hours)	0.5	0.49	1.64	0.63	4.28	0.308
Inotropes	−1.41	4.2	0.24	0	917.13	0.737
ICU stay	−2.76	5.11	0.06	0	1,400.39	0.588
Post-operative complications	3.24	5.29	25.59	0	8,22,159.8	0.54
Time since surgery (months)	−0.15	1.37	0.86	0.06	12.55	0.911

## Discussion

We conducted ADHD questionnaire [ADHD Rating scale IV—Preschool and Connors-3 Short (Parent) form, as per age group] in 98 children aged 3–5 years (18 children) and 6–18 years (80 children). This questionnaire was conducted before cardiac surgery and 13.5 ± 1.2 months after cardiac surgery. All the children included in the study tested negative in their pre-operative questionnaire.

**Primary Objective:** Incidence of ADHD post cardiac surgery.

In our study, the **incidence of ADHD post cardiac surgery was 32.6%**. This is significantly more than the global incidence of ADHD in those with structurally normal hearts (6) and those with congenital heart disease (17). Due to our study design (i.e., prospective), we calculated the incidence of ADHD, although disease burden is better reflected by prevalence and hence the latter is often quoted in literature and guides public health resources.

The prevalence of ADHD post cardiac surgery for congenital heart disease varies from 29% to 44% in literature, which is still more than the global prevalence (6). Yamada et al. (14) cited ADHD prevalence of 29% (SNAP-IV questionnaire) in 112 children aged 7–15 years, who underwent open heart surgery at <1 year of age. Similarly, Sistino et al. (18) calculated a prevalence rate of 44% using Child Health Questionnaire-50 and ADHD- IV survey (home version) in children aged 5–16 years, who had undergone neonatal heart surgery involving repair of aortic arch for Norwood I, interrupted aortic arch and combined repair of aortic coarctation with VSD.

### Questionnaire results

68.7% of those who tested positive for ADHD, did so for inattentiveness, while the rest tested positive for hyperactive + impulsivity. This is similar to findings of Holst et al. (19), who found that in children with TOF, the rate of scores in the clinical range of inattention was almost 6 times more frequent than in the controls and the occurrence was 3 times higher than in children with TGA/VSD. In contrast, clinical range of hyperactive/ impulsivity scores was comparable in children with TGA, VSD and controls; however, our study had a wider variety of CHDs.

**Secondary Objective:** Clinical and Peri-Operative factors affecting the likelihood of ADHD post cardiac surgery.

### Pre-operative factors

We did not find a statistically significant association between gender, age at time of surgery, type of CHD, history of previous cardiac surgery and ADHD positive test result. However, we have summarized the literature review of demographics as follows.

1. **Gender**

We did not find any literature on the sex predilection of ADHD post cardiac surgery for CHD. Studies have shown that ADHD is more common in males (8, 10). Girls are said to be more likely to be diagnosed with the predominantly inattentive type than boys (8), although this finding has not been consistently confirmed in other studies (9). Contrary to this, despite the male predominance in our study population, 68.7% of the positive results of the ADHD questionnaire, was inattentiveness.

2. **Age at time of surgery**

The age group between those who tested positive and negative for ADHD 1 year post cardiac surgery was similar, highlighting that the two groups were comparable in age.

In addition, the final adjusted model of our study highlights that younger age is a robust independent risk factor for ADHD. Younger age at surgery, often necessitated by more complex congenital heart disease, is associated with increased perioperative complexity and risk. This is in discordance with study by Czobor et al, who showed that those operated after 3 years of age had a more severe symptomatology of ADHD, even though those operated earlier had larger number of operations and were more frequently diagnosed with a cyanotic malformation (5).

Further studies are required to explore the importance of age at time of surgery and development of ADHD.

3. **Type of congenital heart disease**

We did not find any association between presence or absence of cyanosis with test result of ADHD questionnaire, which is in accordance with findings of Yamada et al. (14) who also did not find cyanosis to be a positive predictor of ADHD post cardiac surgery.

However, in our study, 39.7% of cyanotics tested positive for ADHD with respect to only 22.5% of acyanotics. 68.7% of those who tested positive for ADHD did so for inattentiveness. This is in accordance with other studies (8, 9) who have found inattentiveness to be more common in cyanotic/complex CHDs (both before and after cardiac surgery). This is likely due to the damage caused by preoperative hypoxemia to the oxygen sensitive prefrontal cortex and corpus striatum, which have been associated with impaired executive control network of attention (13).

4. **History of previous cardiac surgery**

Our findings are in accordance with findings of Yamada et al. (14), who also did not find a positive correlation between total number of cardiac surgeries and likelihood for testing positive for ADHD on SNAP IV scoring. However, anaesthesia has been proposed as a risk factor for development of ADHD, regardless of the procedure performed (15, 16). Unfortunately, there is no global consensus on the ideal cerebroprotective methods of administering anaesthesia.

It is also important to note that negative ADHD questionnaire results were seen in 72.3% of those who did not have a history of previous cardiac surgery, compared to only 57.6% of those who did have the history.

### Operative and intra-operative factors

Operative factors such as cardiopulmonary bypass time, aortic cross clamp time and intra- operative factors (i.e., intra-operative complications) were also evaluated.

1. **Cardiopulmonary Bypass Time-** 10 patients underwent closed heart surgery. Of the rest 88 children, children who tested likely for ADHD had a longer CPB time (73.1 ± 25 min) as compared to those who tested negative (59.1 ± 17.7 min). The difference was statistically significant (*p* value <0.02). This finding is in accordance with multiple studies in literature, including explanations of the pathophysiological basis for the same (5, 13, 14, 20). Sistino et al. (21) explained how the various components of CPB, such as haemodilution (decreased haematocrit and oxygen-carrying capacity), non-pulsatile blood flow, gaseous and particulate micro-emboli and alterations in cerebral blood flow related to carbon dioxide management strategies, subject the cerebral circulation to injury and stress.
2. **Aortic Cross Clamp Time-** 18 patients went on CPB but did not undergo aortic cross clamping. Of the 70 children, those who tested likely to have ADHD post cardiac surgery also had longer aortic cross clamp time (60.6 ± 21.9 min), as compared to those who tested unlikely (41.9 ± 11.4 min). The difference was statistically significant (*p* value <0.001). A study by Czobor et al. showed that those who tested a more severe symptomatology for ADHD had a longer aortic CC time; however, their result was not statistically significant (5).
3. Additionally, those who had a higher inattentive score also had a longer CPB time and aortic CC time, as compared to those who tested as hyperactive + impulsive/nil.

### CPB time/aortic CC time and ADHD

While there are multiple studies in literature that support longer CPB time at a higher risk of developing ADHD, some studies have shown conflicting results (14, 20, 22).

Yamada et al. (14) conducted SNAP IV to screen for ADHD 6–14 years after surgery, and they did not find CPB time or aortic cross-clamp time to be predictive of a higher or “positive” screening score. The authors believed this may be due to an overlooked potential positive predictor, or small sample size (56 children).

Additionally, Shakaya et al. (20) also did not find a statistically significant difference in duration of CPB and aortic CC time between those who had neurodevelopmental delay following repair of cyanotic CHDs. However, they had evaluated children merely 9 months ± 2 weeks after cardiac surgery.

Quartermain et al. (22) conducted neuropsychological tested in children 6 months before and after cardiac surgery for acyanotic heart disease, compared to non-CPB surgical control and nonsurgical control. They found no significant post-operative decline in neuropsychological performance between CPB+ and CPB- groups in the domains of memory, attention, and others. They only found a small but statistically significant difference between CPB+ and CPB- groups in executive function. Hence, while surgery for acyanotic CHD using CPB resulted in variable pattern of neurocognitive changes, but significant deterioration was uncommon and could not be specifically attributed to CPB. These studies challenge our knowledge of the effects of CPB and may help deter our fear in recommending corrective cardiac surgery.

4. **Cut-off for CPB and aortic CC time-** We calculated optimal cut-offs point for CPB and aortic CC time with likelihood of testing positive for ADHD post cardiac surgery. In our study, CPB time >58 min and aortic CC time >46 min was associated with likelihood of a positive test, with sensitivity 83.9% and 85.7% respectively and specificity 56.9% and 63.8% respectively. We did not find any literature corresponding CPB/aortic CC cut-off time with likelihood of developing ADHD post cardiac surgery. Further studies are required to evaluate/validate this finding.
5. **Intra-operative complications-** The presence of intra-operative complication (arrhythmias) did not increase the likelihood of developing ADHD post cardiac surgery (*p* value 0.35). This may be as only 6 patients, i.e., 6.1%, in our study had intra-operative complication. Larger scale studies are required to understand the association between the two. We did not find literature associating intra-operative complications with likelihood of ADHD.
6. **Cooling-** All the patients in our study were cooled 34.4 ± 1.5°Celsius and only one patient was cooled to 25 degrees. As none of the study population underwent deep hypothermic circulatory arrest, nor required ECMO support, hence the effects of the same on risk of developing ADHD post cardiac surgery could not be studied.

### Post-operative factors

Post-operative factors such as duration of mechanical ventilation and inotrope infusion, post- operative complications, requirement of reintervention and duration of hospital stay (including ICU stay) and likelihood of ADHD positivity were evaluated.

1. **Duration of Mechanical Ventilation**

There was a statistically significant association between duration of mechanical ventilation and positive ADHD test score (*p* value 0.021). Studies have shown longer mechanical ventilation duration in those who had a poorer neurodevelopmental outcome (20, 22, 23). Our study also showed that those who tested as hyperactive + impulsive had a longer MV duration (23.8 ± 16.3 h), as compared to those who tested inattentive (18.2 ± 4.3 h)/ negative (15.1 ± 9.4 h).

2. **Duration of Inotropes**

Our study did not find a statistically significant association between duration of inotropes and positive ADHD test score (*p* value 0.464). We did not find studies in literature comparing duration of inotropic support to ADHD specifically. However, our finding is in in accordance with the findings of Shakya et al. (20) who also did not find a statistically significant difference in the inotrope requirement between those who had motor development delay and those who did not, post cardiac surgery for CHD.

3. **Post-operative complications**

Several post-operative complications were seen in 47% of our study population, of which rhythm abnormality was the most common. While 76.5% of the population who did not have any post-operative complication also tested negative for likelihood of ADHD post- surgery, but only 42.6% of the population who had a post-operative complication tested positive for likelihood of ADHD post-surgery. The association between post-operative complication and ADHD was statistically significant (*p* value 0.045). On further evaluation, we determined that they were more likely to be inattentive, as compared to hyperactive + impulsive/Nil. We did not find literature on post-cardiac surgery (for CHD) complications and ADHD.

4. **Requirement of Re-intervention**

Only 4 children included in our study were reintervened after their cardiac surgery, and this was not associated with a positive likelihood on the ADHD questionnaire (*p* value 0.45). However, this may be due to the small sample size and hence needs to be evaluated on larger scale studies. We did not find literature on re-intervention post-cardiac surgery (for CHD) and ADHD. However, in a study by Sarrechia et al. (23), only 2 children required cardiac reintervention, and both had a normal motor DQ on follow up.

5. **Duration of Hospital stay**

Children who tested positive for ADHD had a longer hospital stay, and the difference was small but statistically significant (*p* value <0.001). This is similar to findings of Shakya et al. (20), who also found a difference, albeit not statistically significant. ROC analysis in our study showed that hospital stay >8 days had a good accuracy to predict ADHD likelihood, with sensitivity 68.8% and specificity 72.7%. However, the hospital stay duration in our study comprised of ICU and ward stay, hence we further explored the significance of ICU stay and ADHD test results, which were as follows.

### Duration of ICU stay and ADHD test results

Post-operative ICU stay in the absence of complications varies amongst institutions as per their protocol. Our study shows a shorter ICU stay (2.5 ± 0.9 days) in those who tested negative for ADHD, and the difference was statistically significant (*p* value 0.002). Studies have shown longer ICU stay (ranging from 3 to 12 days) in those who had a poorer neurodevelopmental outcome (20, 22, 23). Our study additionally showed that those who tested as hyperactive had a similar ICU stay (3.6 ± 1.5 days), as compared to those who tested inattentive (3.1 ± 1.2 days), both of which were significantly longer than those who tested negative for ADHD (2.5 ± 0.9 days). Additionally, ROC analysis showed that ICU stay had a fair accuracy to predict ADHD likelihood at value ≥3 days, with sensitivity of 68.8% and specificity of 59.1%.

### Outcome variables

All the participants included in the study were evaluated after 1 year of cardiac surgery. We also found time since surgery did not affect the likelihood of a positive test score for ADHD (*p* value 0.287), although all our study population were evaluated 13.5 ± 1.2 months from their surgery date, which did not have much variation. Some studies in literature have not mentioned time since surgery in their evaluation (5, 19, 23), and others have evaluated for ADHD from 6 months—20 years since surgery (14, 18, 20, 22, 24, 25). Adequate time from cardiac surgery is paramount as previous studies have documented an approximate 50% incidence of transient neuropsychological abnormalities in the early weeks after CPB, anesthesia and pain medication use (22). We chose a 1 year cut off to give adequate time to our patients to return to their normal activities, such as going to school and playing with peers. It is possible that an evaluation too early may miss the diagnosis of ADHD as the child would not have returned to their previous level of activities, while an evaluation too late would increase the morbidity risk due to ADHD.

### Strengths

Our study's strengths lie highlighting the importance of diagnosing ADHD soon after cardiac surgery in children. ADHD is an underdiagnosed and undertreated condition, especially given the mechanism of action of stimulants in a child with heart disease. However, identification, and subsequent prevention, of factors that increase the incidence of ADHD in children post cardiac surgery will help decrease its long-term morbidity. Additionally, statistically significant association with post-operative complications, duration of CPB, aortic CC, mechanical ventilation and subsequent ICU stay, and hospital stay will help us be mindful of these factors.

### Limitations

Our study has some pertinent limitations. Firstly, the follow up period for re-evaluation was arbitrarily chosen as 1 year to conduct the study within the study period. Hence, children with a negative test score could not be re-evaluated. It is possible that the ADHD symptoms occur even later, hence it would be useful to continue to follow the children who test negative and monitor for ADHD symptoms. Some parents continued to be wary of resuming childhood activities, such as school or play, hence some children may still be in home environment. Additionally, there is potential parental bias in overreporting the symptoms.

Secondly, confounding factors such has history of cardiac surgery (long term neurological effects of anesthesia), unequal cyanotics and acyanotics in our study population. parental education and economic status (although the relationship between this and ADHD is controversial) have not been corrected for. Additionally, some children with history of cardiac surgery did not have previous discharge summaries, and hence perioperative details of the previous surgery were lacking. Children who underwent closed heart surgery were also included in the study group but due to small sample size, we could not analyze them separately.

Thirdly, ADHD symptoms were assessed using screening questionnaires, which although are validated but do not establish a formal clinical diagnosis. Although children who screened positive were referred for psychiatric evaluation, the outcomes of these consultations were not systematically captured in the study database, as the primary aim of the study was to evaluate the incidence of ADHD symptoms following cardiac surgery rather than confirm diagnostic status. In addition, the use of a single-informant (parent-report) measure may limit the comprehensiveness of symptom assessment, as it does not capture the child's subjective experience. Future studies should incorporate multi-informant approaches, including self- and teacher-reports, to enhance ecological validity.

Fourthly, the absence of control group in our study hindered us to establish comparative risk and attribute findings specifically to the study population. Future studies including appropriate controls are needed to strengthen causal inferences.

Lastly, our sample size represents a subgroup of the population at risk. Further studies at larger scale are required.

## Conclusion

Our study reveals an incidence rate of 32.6% of ADHD in children 1 year post cardiac surgery for congenital heart disease, which is significantly more than that in general population. We also found a statistically significant association between post- operative complications and testing positive for ADHD. They had a higher inattentive score.

In addition, those who tested positive for ADHD had a statistically significant association with longer CPB time, aortic cross-clamp time, duration of mechanical ventilation, ICU stay, and hospital stay. Children who had a longer CPB time, aortic C-C time and hospital stay had a higher inattentive score. Children who had a longer duration on mechanical ventilation and longer ICU stay had a higher hyperactive + impulsive score.

CPB time >58 min had a good accuracy, aortic cross clamp time >46 min had a very good accuracy, hospital stay >8 days had a good accuracy and ICU stay ≥3 days had a fair accuracy with positive ADHD questionnaire result.

However, we did not find a correlation between potential positive predictors such as history of previous cardiac surgery, presence of cyanosis, intra-operative complications, need for reintervention or inotrope duration and testing positive for ADHD.

Early diagnosis of ADHD with the help of positive predictors will help curtail the morbidities due to ADHD. However, further large-scale studies are required to explore the factors affecting the same.

## Data Availability

The original contributions presented in the study are included in the article/Supplementary Material, further inquiries can be directed to the corresponding author.
